# Speed, agility, and musculoskeletal fitness are independently associated with areal bone mineral density in children

**DOI:** 10.3389/fphys.2023.1080091

**Published:** 2023-02-13

**Authors:** Júlio B. Mello, Fernando Rodríguez-Rodríguez, Luis Gracia-Marco, Juliana L. Teodoro, Anelise R. Gaya, Adroaldo C. A. Gaya

**Affiliations:** ^1^ eFiDac Research Group, School of Physical Education, Pontificia Universidad Católica de Valparaíso, Valparaíso, Chile; ^2^ IRyS Research Group, School of Physical Education, Pontificia Universidad Católica de Valparaíso, Valparaíso, Chile; ^3^ Department of Physical Education and Sports, Faculty of Sport Sciences, Sport and Health University Research Institute (iMUDS), University of Granada, Granada, Spain; ^4^ Instituto de Investigación Biosanitaria ibs.GRANADA, Granada, Spain; ^5^ Centro de Investigación Biomédica en Red Fisiopatología de la Obesidad y Nutrición (CIBERobn), Instituto de Salud Carlos III, Madrid, Spain; ^6^ Universidade Federal do Rio Grande do Sul, Post-graduate program of movement Science, Porto Alegre, Brazil

**Keywords:** physical fitness, bone tissue, physical conditioning, school, child

## Abstract

**Background:** There is still little understanding of the associations between physical fitness variables and bone health in children taking into account key confounders.

**Aim:** The aim of this study was to analyze the associations between performance in tests of speed, agility, and musculoskeletal fitness (power of the upper and lower limbs) with bone mass of different regions in children, considering the adjustment to maturity-offset, lean percentage, and sex.

**Methods:** Cross-sectional study design: the sample consisted of 160 children aged 6–11 years. The physical fitness variables tested were 1) speed, assessed with the running test at a maximum speed of 20 m; 2) agility, assessed through the 4×4-m square test; 3) lower limb power, assessed using the standing long jump test, and 4) upper limb power, assessed using the 2-kg medicine ball throw test. Areal bone mineral density (aBMD) was obtained from the analysis of body composition by dual-energy X-ray absorptiometry (DXA). Simple and multiple linear regression models were performed using the SPSS software.

**Results:** In the crude regression analyses, the results indicated a linear relationship between all the physical fitness variables and aBMD in all body segments, but maturity-offset, sex, and lean mass percentage seemed to have an effect on these relationships. Except for the upper limb power, the other physical capacities (speed, agility, and lower limb power) were associated with aBMD in at least three body regions in the adjusted analyses. These associations occurred in the spine, hip, and leg regions, and the aBMD of the legs presented the best association magnitude (*R* ^2^).

**Conclusion:** There is a significant association between speed, agility, and musculoskeletal fitness, specifically the lower limb power and aBMD. That is, the aBMD is a good indicator of the relationship between fitness and bone mass in children, but it is essential to consider specific fitness variables and skeletal regions.

## Introduction

The bone mass acquisition during adolescence is related to several factors [e.g., genetic (Slemenda et al., 2009), maturational (Theintz et al., 1992), and lifestyle (Bachrach, 2001)]. However, there is evidence that sex hormones are important modulators of bone mass in the period of maturation (in other periods too), suggesting an estrogenic effect on the trabecular and cortical bone (Arabi et al., 2004; Yilmaz et al., 2005). Thus, it is assumed that optimizing peak bone mass in childhood and adolescence may be the main strategy to compensate for the decline in bone density associated with advancing age and to promote long-term bone health (Weaver et al., 2016).

In this sense, the systematic reviews by Mello et al. (2021) and García-Hermoso et al. (2021) demonstrate that interventions with physical exercise and physical activity with vigorous intensity (both with a positive effect on physical fitness) promote gains in bone mass in children and adolescents [e.g., bone mineral content (BMC) and bone mineral density (BMD)]. As Gómez-Bruton et al. (2017) have shown that increase in lower limb power through plyometric exercises (effects on muscle power) in children and adolescents causes positive effects on general BMC.

However, it is noteworthy that the strong literature data are about the cause–effect relationship between physical activity interventions and bone mass, called the osteogenic effect (Gómez-Bruton et al., 2017; García-Hermoso et al., 2021; Mello et al., 2021). Some longitudinal studies (Kemper, 2000; Vicente-Rodríguez et al., 2008) have already demonstrated that in the long term, some physical capacities that are developed from activities with constant impacts, strong muscle contractions, and constant osteotendinous tensions may be associated with osteogenesis. The physical capacities described in the longitudinal studies are musculoskeletal fitness (i.e., muscular strength, power, and endurance), agility, and speed.

Recently, Gómez-Bruton et al. (2020) investigated the association between global physical fitness (such as cardiorespiratory fitness, agility, speed, and musculoskeletal fitness) and bone health indicators (BMD in different body sites, e.g., the spine, hip, and neck femur) in a sample of 92 Spanish children aged between 3 and 5 years. The results indicated that relative upper and lower limb muscle strength and speed/agility predicted all measured bone variables, except for BMD. The authors also point out that the global physical fitness results are determinants for bone structure and strength and that, consequently, the performance of physical fitness tests could provide useful information related to bone health in children. It should be noted that although these children are very young, the associations can be expected in the other stages of childhood as well due to the uninterrupted process of bone growth.

Thus, experimental evidence points to a possible relationship between physical fitness and bone health indicators in childhood. But this possible relationship is not clear when we talk about children and adolescents in the growth peak for example. Some studies have strongly suggested that musculoskeletal fitness, mainly power and strength, is related to good bone health (evidence from the osteogenic effect of exercises) (Kemper et al., 2000; Gracia-Marco et al., 2011; Janz et al., 2015; Gómez-Bruton et al., 2017; Gómez-Bruton et al., 2020; Henriques-Neto et al., 2020). Although there is less evidence, a review (Mello et al., 2021) has also indicated the relationship between speed and agility with bone health—specifically with the hip and spine bones—and development in children, however it had not evaluated the influence of maturation.

Therefore, the aforementioned studies suggest that there is still little understanding of the associations between physical fitness variables and bone health indicators in children and adolescents; however, they are promising in pointing out some directions. In this sense, the aim of this study is to analyze the associations between performance in tests of speed, agility, and musculoskeletal fitness (power of the upper and lower limbs) and the bone mass of different regions of the body in children, considering the adjustment to maturity-offset, lean and fat percentage, and sex.

## Materials and methods

### Study design

This cross-sectional study with a quantitative approach and non-probabilistic sample was carried out after approval of the Universidade Federal do Rio Grande do Sul Research Ethics Committee (number: 3.414.512; Brazilian system CAAE: 12222019.9.0000.5347).

### Research subjects and data collection procedure

The sample consisted of 160 children aged 6–11 years from the first to fifth year of the public elementary school in the city of Porto Alegre, Brazil. To identify the test’s power from the sample size of 160 children, *a posteriori* sample calculation was performed using the G-Power version 3.1 program, and for this, the equation directed to the proposed association test was used. The calculation was performed for tests of the F family, considering that the research project foresees the association analyses those were conducted in another study (Mello et al., 2021). The alpha used was 0.05; the used effect size for differences was f^2^ = 0.15 (moderate); and eight variables were used as predictors. From this protocol, considering the sample size of the present study, the power of the test (1 − β) was identified as 0.95.

For data collection, contact was made with the school. After signing the authorization terms (school authorization form), all the children who were enrolled from the first to fifth year of the elementary school received an invitation to participate in the research with the consent terms (parents/guardian and children consent and assent forms). After this stage, a class period was scheduled to carry out the physical tests. The dual-energy X-ray absorptiometry (DXA) exam was performed at the Exercise Research Laboratory (LAPEX) at the Universidade Federal do Rio Grande do Sul, Brazil. For this purpose, an evaluation time was scheduled with the parents/guardians. All tests and examinations were performed by previously trained researchers. This procedure was conducted at the beginning of the academic years 2017 and 2018.

### Physical fitness

The procedures for collecting physical fitness variables were performed according to the PROESP-BR (*Projeto Esporte Brasil*) Guidelines for Measurements, Tests, and Assessments (Gaya et al., 2021) and have been described in detail in previous studies (Mello et al., 2015; Mello et al., 2016; Pedretti et al., 2020). The physical fitness variables tested were 1) sprint, assessed with the running test at a maximum speed of 20 m; 2) agility, assessed through the 4×4-m square test; 3) lower limb power (LLP), assessed using the standing long jump test, and 4) upper limb power (ULP), assessed using the 2-kg medicine ball throw test. These tests have international use and validation with good evidence (Bös and Schlenker, 2011; Calleja-González et al., 2015) and are widely used in Brazil (Pedretti et al., 2020).

### Bone outcomes

Areal bone mineral density (aBMD) was collected from the analysis of body composition according to the recommendations of the manufacturer of the DXA device of the GE Healthcare model, Lunar Prodigy (Madison, United States). A trained researcher and qualified laboratory technician carried out the examinations and handling of the device, respectively. The device was calibrated once a day before the evaluation sessions. Children were instructed to remove any metal material and wear clothes without zips, buckles, or buttons. Before the evaluation, the guardians were instructed that DXA uses X-rays emitted at two different energy levels to allow for distinguishing the bone tissue from its surrounding soft tissue and that radiation exposure is low with DXA (less than 5 mrem/scan). The evaluator placed the subjects in the supine position and asked them to remain motionless during the measurement, for approximately 5 min, while the equipment arm passed over the body in the head–foot direction. The values were automatically calculated using the equipment’s software (Encore version 14.1, Madison, United States). The values of aBMD (e.g., 0.978 g/cm^2^) have been described for the total body, total body less the head, and body segments such as the trunk, spine, arms, hip, and legs.

### Covariates

Due to the influence that bone mass indicators suffer from biological variables (Bachrach, 2001), the lean mass percentage and maturity-offset were considered covariates. The total body lean mass percentages were made available during the DXA exam, along with the bone variables. For maturity-offset calculation, the following variables were required: height, body mass, sitting height, and length of the lower limbs.

The data collection for these variables and the calculation of maturity offset followed the recommendations proposed by Mirwald et al. (2002). The children performed the measurements in light clothes (e.g., physical education class clothes) and without shoes. All anthropometric procedures followed are as described in PROESP-BR Guidelines for Measurements, Tests, and Assessments (Gaya et al., 2021). For the measurement of body mass, a portable scale with an accuracy of up to 100 g was used. During the assessment, the children and adolescents remained standing with their elbows extended and close to their bodies. For the measurement of height and sitting height, a portable stadiometer or measuring tape with a precision of up to 2 mm was used. For the sitting height, a bench with a standard size of 40 cm was used, and the zero point of the measuring tape was on the bench.

### Data analysis

For the treatment of data, a descriptive analysis was first performed. In this analysis, the mean value and standard deviation were identified for continuous variables. In the next step, an exploratory analysis was conducted to identify the normality parameters in the physical fitness variables. In this procedure, the Kolmogorov–Smirnov test was performed.

After these steps, the association analyses were performed to estimate variabilities of bone health indicators (outcomes) from the physical fitness variables (analyzed separately). At this stage, a correlation matrix was created between the physical fitness variables and all the bone variables; this procedure is a prerequisite for linear regression. Then, simple and multiple linear regression equations were used. Collinearity was checked for the variables using the variance inflation factor (VIF) and tolerance levels. In the multiple regression analysis, the equation was adjusted for covariates (sex, maturity-offset, and lean mass percentage) after the multicollinearity test. For these analyses, we have applied the Bonferroni correction for multiple testing and assumed a significant *p*-value below 0.007, which results from dividing the alpha value (0.05) by the number of dependent variables, in our case 7. All statistical analyses were performed using the SPSS for Windows version 24.0. For all analyses, an alpha value of 0.05 was considered.

## Results

### Descriptive analysis and correlation

Table 1 describes information on the distribution and central tendency of anthropometric variables, maturity-offset, physical fitness, lean mass percentage, age, and aBMD in each body region. The mean values of the variables age, height, and weight were similar in the total sample and when stratified by sex. The other variables had different mean values.

**TABLE 1:** Distribution and central tendency of anthropometric variables, maturity-offset, physical fitness, lean and fat percentage, age and aBMD.

	Total		Boys		Girls	
	n	Ẋ ± SD	n	Ẋ ± SD	n	Ẋ ± SD
Anthropometry, maturity offset, and physical fitness						
Age (year)	160	8.90 ± 1.50	85	8.20 ± 1.60	75	8.30 ± 1.50
Height (m)	155	1.33 ± 0.10	85	1.34 ± 0.10	74	1.33 ± 0.11
Weight (kg)	155	33.03 ± 10.14	85	32.80 ± 9.31	74	33.29 ± 11.03
Maturity offset (year)	152	−3.02 ± 1.73	79	−4.01 ± 1.11	73	−1.95 ± 1.65
LM%	160	21.73 ± 4.84	85	22.27 ± 4.31	75	21.12 ± 5.34
Sprint (s)	154	4.54 ± 0.85	80	4.45 ± 0.96	74	4.63 ± 0.70
Agility (s)	153	7.91 ± 1.00	79	7.70 ± 1.01	74	8.13 ± 0.94
Lower limb power (cm)	154	110.58 ± 24.04	80	117.09 ± 24.57	74	103.54 ± 21.48
Upper limb power (cm)	152	184.23 ± 53.85	79	191.92 ± 51.83	73	175.90 ± 55.10
Bone mass variables						
aBMD (g/cm^2^)						
Total body	159	0.847 ± 0.091	84	0.849 ± 0.086	75	0.845 ± 0.096
TBLH	159	0.717 ± 0.098	84	0.714 ± 0.089	75	0.722 ± 0.108
Arms	159	0.559 ± 0.075	84	0.555 ± 0.072	75	0.565 ± 0.078
Trunk	160	0.677 ± 0.090	85	0.670 ± 0.077	75	0.684 ± 0.103
Spine	160	0.728 ± 0.100	85	0.718 ± 0.081	75	0.739 ± 0.116
Hip	160	0.715 ± 0.106	85	0.706 ± 0.094	75	0.724 ± 0.118
Legs	160	0.831 ± 0.124	85	0.832 ± 0.117	75	0.830 ± 0.133

n, sample number; Ẋ ± SD, mean and standard deviation; m, meter; kg, kilogram; LM%, lean mass percentage; aBMD, areal bone mineral density; TBLH, total body less head.

The correlation between the physical fitness variables and bone mass variables is described in Table 2. Several associations showed minimal correlation (r < 0.5); however, due to the lack of regularity between the associations, all bone variables were included in the linear regression analyses. Regarding collinearity, there was a strong correlation between age and maturity-offset (r > 0.7), thus the multiple analyses were adjusted only by sex, lean and fat percentage, and maturity-offset.

**TABLE 2:** Correlation matrix between physical fitness and bone mass variables.

	Sprint		Agility		Lower limb power		Upper limb power	
	r	*p*-value	r	*p*-value	r	*p*-value	r	*p*-value
**aBMD**								
Total body	−0.54	<0.001	−0.47	<0.001	0.36	<0.001	0.56	<0.001
TBLH	−0.52	<0.001	−0.44	<0.001	0.30	<0.001	0.60	<0.001
Arms	−0.38	<0.001	−0.30	<0.001	0.25	0.001	0.45	<0.001
Trunk	0.44	<0.001	0.37	<0.001	0.21	0.007	0.55	<0.001
Spine	−0.35	<0.001	−0.29	0.002	0.10	0.200	0.50	<0.001
Hip	−0.51	<0.001	−0.41	<0.001	0.30	<0.001	0.52	<0.001
Legs	−0.58	<0.001	−0.50	<0.001	0.37	<0.001	0.63	<0.001

r, correlation value; *p*-value, value of statistical significance; aBMD, areal bone mineral density; TBLH, total body less head.

### Association analysis—simple and multiple regression

#### Sprint

The association of sprint with aBMD presented a magnitude that varied between −0.043 (arms) and −0.108 (legs) in the crude analyses (Table 3). When the co-variables were included in the analyses (adjustment), some variables lost their statistical significance. Considering the constant co-variables, the sprint was associated with aBMD in the total body (for every 1 s more in the 20 m run test, a 0.028 g/cm^2^ reduction in the aBMD was estimated), total body less head (0.021 g/cm^2^ of reduction per second), hip (0.032 g/cm^2^ of reduction per second), and legs (0.035 g/cm^2^ of reduction per second). The value of the coefficient of determination (*R* ^2^) in all analyses was high (>65), indicating that sprint, sex, lean mass, and maturity-offset forms an important set of variables to explain the variability of aBMD in some body regions.

**TABLE 3:** Estimation of the variability of aBMD in different body segments from sprint and covariates.

	Crude analysis			Adjusted analysis*			
	*Β*	95% CI	*p*-value	*R* ^2^	β	95% CI	*p*-value
**aBMD**							
Total body	−0.074	−0.093 to −0.056	**<0.001**	0.67	−0.028	−0.043 to −0.012	**0.001**
TBLH	−0.078	−0.098 to −0.057	**<0.001**	0.78	−0.021	−0.034 to −0.007	**0.004**
Arms	−0.043	−0.059 to −0.026	**<0.001**	0.52	−0.007	−0.022 to −0.008	0.374
Trunk	−0.061	−0.081 to −0.041	**<0.001**	0.69	−0.011	−0.026 to −0.004	0.149
Spine	−0.053	−0.076 to −0.030	**<0.001**	0.61	−0.001	−0.019 to 0.019	0.978
Hip	−0.082	−0.104 to −0.060	**<0.001**	0.67	−0.032	−0.050 to −0.013	**0.001**
Legs	−0.108	−0.133 to −0.083	**<0.001**	0.80	−0.035	−0.051 to −0.018	**<0.001**

^*^ *R* ^2^: adjusted coefficient of determination by sex, lean percentage, and maturity offset.

*β*, regression unstandardized coefficient value; 95% CI, confidence interval of 95%; *p*-value, value of statistical significance; aBMD, areal bone mineral density; TBLH, total body less head.

*p*-values below 0.007 were considered as significant after applied Bonferroni correction for multiple testing.

#### Agility

The association of agility with aBMD presented a magnitude that varied between −0.023 (arms) and −0.064 (legs) in the crude analyses (Table 4). When the co-variables were included in the analyses (adjustment), some variables lost their statistical significance. Considering the constant co-variables, agility was associated with aBMD in the total body (for every 1 s more in the 4×4-m square test, a 0.014 g/cm^2^ reduction in aBMD was estimated) and legs (0.020 g/cm^2^ of reduction per second). The value of the coefficient of determination (*R* ^2^) in all analyses was high (>65), indicating that agility, sex, lean mass, and maturity-offset forms an important set of variables to explain the variability of aBMD in some body regions.

**TABLE 4:** Estimation of the variability of aBMD in different body segments from the agility and covariates.

	Crude analysis			Adjusted analysis*			
	β	95% CI	*p*-value	*R* ^2^	Β	95% CI	*p*-value
**aBMD**							
Total Body	−0.043	−0.056 to −0.030	**<0.001**	0.65	−0.014	−0.025 to −0.004	**0.006**
TBLH	−0.044	−0.058 to −0.030	**<0.001**	0.77	−0.010	−0.019 to −0.001	0.034
Arms	−0.023	−0.034 to −0.011	**<0.001**	0.51	−0.001	−0.011 to 0.009	0.859
Trunk	−0.034	−0.048 to −0.020	**<0.001**	0.69	−0.004	−0.014 to 0.005	0.388
Spine	−0.030	−0.046 to −0.014	**<0.001**	0.59	0.000	−0.012 to 0.003	0.947
Hip	−0.045	−0.060 to −0.029	**<0.001**	0.64	−0.013	−0.025 to 0.001	0.037
Legs	−0.064	−0.081 to −0.047	**<0.001**	0.79	−0.020	−0.030 to −0.009	**<0.001**

*R ^2^, adjusted coefficient of determination by sex, lean percentage, and maturity-offset.

*β*, regression unstandardized coefficient value; 95% CI, confidence interval of 95%; *p*-value, the value of statistical significance; aBMD, areal bone mineral density; TBLH, total body less head.

*p*-values below 0.007 were considered as significant after applying Bonferroni correction for multiple testing.

#### Lower limb power

The association of lower limb power with aBMD presented a magnitude that was −0.001 for all body regions, except for the legs (−0.002 for legs) in the crude analyses (Table 5). When the co-variables were included in the analyses (adjustments), some variables lost their statistical significance. Considering the constant co-variables, the lower limb power was associated with aBMD in the legs (for every 1 cm more in the standing long jump test, it was estimated to increase by 0.001 g/cm^2^ of aBMD). The value of the coefficient of determination (*R* ^2^) in all analyses was high (>60), indicating that lower limb power, sex, lean mass, and maturity-offset forms an important set of variables to explain the variability of aBMD in some body regions.

**TABLE 5:** Estimation of the variability of aBMD in different body segments from the lower limb power and covariates.

	Crude analysis			Adjusted analysis*			
	β	95% CI	*p*-value	*R* ^2^	β	95% CI	*p*-value
**aBMD**							
Total body	0.001	0.001–0.002	**<0.001**	0.64	<0.001	0.000–0.001	0.032
TBLH	0.001	0.001–0.002	**<0.001**	0.77	<0.001	0.000–0.001	0.178
Arms	0.001	0.001–0.002	**<0.001**	0.51	<0.001	0.000–0.001	0.407
Trunk	0.001	0.000–0.001	**0.007**	0.69	<0.001	0.000–0.001	0.554
Spine	0.001	0.000–0.001	0.217	0.62	0.001	0.000–0.001	0.018
Hip	0.001	0.001–0.002	**<0.001**	0.65	<0.001	0.000–0.001	0.114
Legs	0.002	0.001–0.003	**<0.001**	0.72	0.001	0.001–0.002	**0.006**

*R ^2^, adjusted coefficient of determination by sex, lean percentage, and maturity offset.

*β*, regression unstandardized coefficient value; 95% CI, confidence interval of 95%; *p*-value, value of statistical significance; aBMD, areal bone mineral density; TBLH, total body less head.

*p*-values below 0.007 were considered as significant after applied Bonferroni correction for multiple testing.

#### Upper limb power

The association of upper limb power with aBMD presented a magnitude that was −0.001 for all body regions in crude analyses (Table 6). When the co-variables were included in the analyses (adjustments), all variables lost their statistical significance.

**TABLE 6:** Estimation of the variability of aBMD in different body segments from the upper limb power and covariates.

	Crude analysis			Adjusted analysis*			
	β	95% CI	*p*-value	*R* ^2^	β	95% CI	*p*-value
**aBMD**							
Total body	0.001	0.001–0.001	**<0.001**	0.64	0.000	0.000–0.001	0.285
TBLH	0.001	0.001–0.001	**<0.001**	0.77	0.000	0.000–0.001	0.198
Arms	0.001	0.001–0.001	**<0.001**	0.50	0.000	0.000–0.000	0.994
Trunk	0.001	0.001–0.001	**<0.001**	0.69	0.000	0.000–0.000	0.319
Spine	0.001	0.001–0.001	**<0.001**	0.60	0.000	0.000–0.001	0.479
Hip	0.001	0.001–0.001	**<0.001**	0.65	0.000	0.000–0.001	0.567
Legs	0.001	0.001–0.002	**<0.001**	0.78	0.000	0.000–0.001	0.075

*R ^2^, adjusted coefficient of determination by sex, lean percentage, and maturity-offset.

*β*, regression unstandardized coefficient value; 95% CI, confidence interval of 95%; *p*-value, value of statistical significance; aBMD areal bone mineral density; TBLH, total body less head.

*p*-values below 0.007 were considered as significant after applied Bonferroni correction for multiple testing.

## Discussion

The aim of this study was to analyze the associations between performance in tests of speed, agility, and musculoskeletal fitness (power of upper and lower limbs) with the bone mass of different regions of the body in children, considering the adjustment to maturity-offset, lean mass percentage, and sex. Thus, the main evidence of this study points to a relationship between speed, agility, and lower limb power, the capacities those require constant ground impact plus large muscle contraction, with the aBMD in different skeletal regions (TBLH, spine, hip, and specially legs) of children regardless of the level of lean mass, sex, and distance to the peak of their growth.

For both speed and agility, it was not possible to clearly observe associations in the correlation matrix. However, in these results, the highest correlations (later confirmed in the regression models) were with the aBMD of the legs. This relationship seems logical since the greatest muscle demand and the greatest bone overload for sprint and agility activities are in the legs (Douma-van Riet et al., 2012).

The physical activities that potentially develop speed and agility are high-intensity running (with or without changing the direction). Earlier studies (Castro-Piñero et al., 2010; Chaouachi et al., 2014) have indicate that children who have higher performances in different running tests are those who routinely practice activities with this characteristic. In this way, as Gómez-Bruton et al. (2017) have already demonstrated, our results confirm that activities that have a high volume of stress on the bones (high-intensity running) seem to be beneficial for their development.

Running activities are characterized by a succession of jumps, and the ground reaction force directly impacts the bones with each touch of the foot on the ground (Završnik et al., 2017). The force impressed on the ground by a child during a high-intensity run is approximately two–three times their body weight (Anliker et al., 2011). In this sense, a force passes through the bones of the legs, hips, and lumbar spine specifically, causing the piezoelectric effect (passage of electric currents through the interior of the bones). Evidence indicates that the piezoelectric effect is one of the main reasons responsible for the osteogenic effect of these activities (Theintz et al., 1992).

In the same sense, the lower limb power was strongly related to all bone variables on the basis of the correlation analyses. In the same logic, the muscular power of the lower limbs mainly responds to the activities of different jumps, which also take advantage of the benefits of the ground reaction force (jumps can have 3.5–5 body weights in ground reaction force (Gómez-Bruton et al., 2017). However, for this physical capacity specifically, rapid muscle contraction is more required that causes the tendons to also overload the bones, thereby enhancing the osteogenic effect.

Although lower limb power mainly responds to activities with higher osteogenic potential, when the associations were adjusted for sex, maturation, and lean mass, only the aBMD of the spine, total body, and legs remained associated. This demonstrates that although there is a tendency for more powerful children to have a better profile of bone mass and lean mass, the maturational stage has to be evaluated as well. Our suggestion for future studies is that this association can be explored by considering mediation and moderation analyses of these covariates.

The physical capacity that had lost the significant association, after adjustment, was the power of the upper limbs. As discussed earlier, we expected that because of the traction exerted by the tendons, the associations would also occur with this variable. However, we understand that the test does not completely isolate muscle power and may mechanically benefit children and adolescents with longer, but not necessarily more powerful, arms (Ikeda et al., 2009).

Furthermore, the evident lack of activities that generate a continuous impact on the upper limbs may be the explanation for the results found. Studies with adolescent fighters showed that the BMD was higher than that in non-fighters (Ciaccioni et al., 2019), mainly due to the characteristics of the activities performed. Furthermore, lean mass, as found in other studies (Vicente-Rodríguez et al., 2008; Anliker et al., 2011), influenced the power performance level of children and adolescents, a factor that was not considered in the present study.

This study has strong points that deserve to be highlighted. The evidence presented comes largely from the gold standard assessments. Furthermore, the physical tests that were used have great national repercussion and have acceptable validity criteria (see the Materials and methods section). Furthermore, as far as we know, the analysis proposal has not yet been presented for the age group studied, making the study unprecedented. However, it is important that the readers’ interpretations are carried out knowing the limitations of this study. The development of physical fitness is different in boys and girls, and the ideal was a stratified analysis; however, the sample size did not allow us to do this. The maturity-offset proposed by Mirwald et al. (2002) does not include children aged between 6 and 7 years, while in this case, the maturity-offset is just an estimate and have to be analyzed carefully. Furthermore, more specific bone mass assessments such as the femoral neck and radioulnar epiphysis would be very appropriate for children, but the DXA device used did not have specific software for this assessment. We found that the adjustment analysis could have the considered physical activity levels but could not access this variable in all participants.

## Conclusion

Collectively, the results indicated that speed, agility, and musculoskeletal fitness, specifically lower limb power, are associated with aBMD in different body regions. These associations occur in the total body, spine, hip, and legs when adjusted for sex, maturity-offset and lean body mass, and aBMD of the legs having the best magnitude of association (*R* ^2^) with these three physical fitness components. Furthermore, sprint ability appears to be associated with both legs and hip aBMD, being a great predictor of bone health. In summary, the aBMD is a good indicator between fitness and bone mass relationship in children, but it is important to consider what fitness variable and what skeletal region.

## Data Availability

The original contributions presented in the study are included in the article/Supplementary Materials, further inquiries can be directed to the corresponding author.

## References

[B1] Anliker E. Rawer R. Boutellier U. Toigo M. (2011). Maximum ground reaction force in relation to tibial bone mass in children and adults. Med. Sci. Sports Exerc 43, 2102–2109. 10.1249/MSS.0b013e31821c4661 21502901

[B2] Arabi A. Nabulsi M. Maalouf J. Choucair M. Khalife H. Vieth R. (2004). Bone mineral density by age, gender, pubertal stages, and socioeconomic status in healthy Lebanese children and adolescents. Bone 35, 1169–1179. 10.1016/j.bone.2004.06.015 15542043

[B3] Bachrach L. K. (2001). Acquisition of optimal bone mass in childhood and adolescence. Trends Endocrinol Metab 12, 22–28. 10.1016/s1043-2760(00)00336-2 11137037

[B4] Bös K. Schlenker L. (2011). “Deutscher motorik-test 6–18 (DMT 6–18),” in Bildung im Sport (Wiesbaden: VS Verlag für Sozialwissenschaften), 337–355.

[B5] Calleja-González J. J. Los Arcos A. Gaizka M. Casamichana D. San Roman-Quintana J. Yanci J. (2015). Reproducibilidad de test de aceleración y cambio de dirección en fútbol. [Reproducibility of test acceleration and change of direction in football]. RICYDE 11, 104–115. 10.5232/ricyde2015.04001

[B6] Castro-Piñero J. González-Montesinos J. L. Mora J. Sjostrom M. Ruiz J. R. (2010). Percentile values for running sprint field tests in children ages 6-17 years: Influence of weight status. Res. Q. Exerc Sport 81, 143–151. 10.1080/02701367.2010.10599661 20527299

[B7] Chaouachi A. Othman A. Hammami R. Drinkwater E. J. Behm D. G. (2014). The combination of plyometric and balance training improves sprint and shuttle run performances more often than plyometric-only training with children. J. Strength Cond. Res. 28, 401–412. 10.1519/JSC.0b013e3182987059 23669821

[B8] Ciaccioni S. Condello G. Guidotti F. Capranica L. (2019). Effects of judo training on bones: A systematic literature review. J. Strength Cond. Res. 33, 2882–2896. 10.1519/JSC.0000000000002340 29239994

[B9] Douma-van Riet D. Verschuren O. Jelsma D. Kruitwagen C. Smits-Engelsman B. Takken T. (2012). Reference values for the muscle power sprint test in 6- to 12-year-old children. Pediatr. Phys. Ther. 24, 327–332. 10.1097/PEP.0b013e3182694a4c 22965203

[B10] García-Hermoso A. Ezzatvar Y. Ramírez-Vélez R. Olloquequi J. Izquierdo M. (2021). Is device-measured vigorous physical activity associated with health-related outcomes in children and adolescents? A systematic review and meta-analysis. J. Sport Health Sci. 10, 296–307. 10.1016/j.jshs.2020.12.001 33285309 PMC8167335

[B11] Gaya A. R. Gaya A. Pedretti A. (2021). “Projeto Esporte Brasil, PROESP-Br: Manual de medidas, testes e avaliações. 2021st,” in Porto Alegre (UFRGS/ESEFID).

[B12] Gómez-Bruton A. Marín-Puyalto J. Muñiz-Pardos B. Lozano-Berges G. Cadenas-Sanchez C. Matute-Llorente A. (2020). Association between physical fitness and bone strength and structure in 3- to 5-year-old children. Sports Health 12, 431–440. 10.1177/1941738120913645 32442050 PMC7485023

[B13] Gómez-Bruton A. Matute-Llorente Á González-Agüero A. Casajus J. A. Vicente-Rodriguez G. (2017). Plyometric exercise and bone health in children and adolescents: A systematic review. World J. Pediatr. 13, 112–121. 10.1007/s12519-016-0076-0 28101776

[B14] Gracia-Marco L. Vicente-Rodríguez G. Casajús J. A. Molnar D. Castillo M. J. Moreno L. A. (2011). Effect of fitness and physical activity on bone mass in adolescents: The HELENA study. Eur. J. Appl. Physiol. 111, 2671–2680. 10.1007/s00421-011-1897-0 21394637

[B15] Henriques-Neto D. Magalhães J. P. Hetherington-Rauth M. Santos D. A. Baptista F. Sardinha L. B. (2020). Physical fitness and bone health in young athletes and nonathletes. Sports Health 12, 441–448. 10.1177/1941738120931755 32660392 PMC7485020

[B16] Ikeda Y. Miyatsuji K. Kawabata K. Fuchimoto T. Ito A. (2009). Analysis of trunk muscle activity in the side medicine-ball throw. J. Strength Cond. Res. 23, 2231–2240. 10.1519/JSC.0b013e3181b8676f 19826303

[B17] Janz K. F. Thomas D. Q. Ford M. A. Williams S. M. (2015). Top 10 research questions related to physical activity and bone health in children and adolescents. Res. Q. Exerc Sport 86, 5–12. 10.1080/02701367.2014.995019 25664669

[B18] Kemper H. C. G. (2000). Skeletal development during childhood and adolescence and the effects of physical activity. Pediatr. Exerc Sci. 12, 198–216. 10.1123/pes.12.2.198

[B19] Kemper H. C. G. Twisk J. W. R. van Mechelen W. Post G. B. Roos J. C. LiPs P. (2000). A fifteen-year longitudinal study in young adults on the relation of physical activity and fitness with the development of the bone mass: The amsterdam growth and health longitudinal study. Bone 27, 847–853. 10.1016/s8756-3282(00)00397-5 11113397

[B20] Mello J. B. Hernandez M. S. Farias V. M. Pinheiro E. Bergmann G. (2015). Aptidão física relacionada ao desempenho motor de Adolescentes de Uruguaiana, Rio Grande do Sul. Rev. Bras. Cien Mov. 23, 72–79. 10.18511/0103-1716/rbcm.v23n4p72-79

[B21] Mello J. B. Nagorny G. A. K. Haiachi M. D. C. Gaya A. R. Gaya A. C. A. (2016). Projeto Esporte Brasil: Perfil da aptidão física relacionada ao desempenho esportivo de crianças e adolescentes. Braz J. Kinan Hum. Perf. 18, 658. 10.5007/1980-0037.2016v18n6p658

[B22] Mello J. B. Pedretti A. García-Hermoso A. Martins C. M. L. Gaya A. R. Duncan M. J. (2021). Exercise in school Physical Education increase bone mineral content and density: Systematic review and meta-analysis. Eur. J. Sport Sci. 22, 1618–1629. 10.1080/17461391.2021.1960426 34328066

[B23] Mirwald R. L. Baxter-Jones G. A. D. Bailey D. A. Beunen G. P. (2002). An assessment of maturity from anthropometric measurements. Med. Sci. Sports Exerc 34, 689–694. 10.1097/00005768-200204000-00020 11932580

[B24] Pedretti A. Mello J. B. Gaya A. R. Cezar Araujo Gaya A. (2020). Health- and skill-related physical fitness profile of Brazilian children and adolescents: A systematic review. Rev. Bras. Ativ. Fís Saúde 25, 1–10. 10.12820/rbafs.25e0131

[B25] Slemenda C. W. Christian J. C. Williams C. J. Norton J. A. Johnston C. C. (2009). Genetic determinants of bone mass in adult women: A reevaluation of the twin model and the potential importance of gene interaction on heritability estimates. J. Bone Min. Res. 6, 561–567. 10.1002/jbmr.5650060606 1887818

[B26] Theintz G. Buchs B. Rizzoli R. Slosman D. Clavien H. Sizonenko P. C. (1992). Longitudinal monitoring of bone mass accumulation in healthy adolescents: Evidence for a marked reduction after 16 years of age at the levels of lumbar spine and femoral neck in female subjects. J. Clin. Endocrinol. Metab. 75, 1060–1065. 10.1210/jcem.75.4.1400871 1400871

[B27] Vicente-Rodríguez G. Urzanqui A. Mesana M. I. Ortega F. B. Ruiz J. R. Ezquerra J. (2008). Physical fitness effect on bone mass is mediated by the independent association between lean mass and bone mass through adolescence: A cross-sectional study. J. Bone Min. Metab. 26, 288–294. 10.1007/s00774-007-0818-0 18470671

[B28] Weaver C. M. Gordon C. M. Janz K. F. Kalkwarf H. J. Lappe J. M. Lewis R. (2016). The national osteoporosis foundation’s position statement on peak bone mass development and lifestyle factors: A systematic review and implementation recommendations. Osteoporos. Int. 27, 1281–1386. 10.1007/s00198-015-3440-3 26856587 PMC4791473

[B29] Yilmaz D. Ersoy B. Bilgin E. Gumuser G. Onur E. Pinar E. D. (2005). Bone mineral density in girls and boys at different pubertal stages: Relation with gonadal steroids, bone formation markers, and growth parameters. J. Bone Min. Metab. 23, 476–482. 10.1007/s00774-005-0631-6 16261455

[B30] Završnik J. Pišot R. Šimunič B. Kokol P. Blazun Vosner H. (2017). Biomechanical characteristics of skeletal muscles and associations between running speed and contraction time in 8- to 13-year-old children. J. Int. Med. Res. 45, 231–245. 10.1177/0300060516687212 28222639 PMC5536606

