# Supplementary material for: Speed, agility, and musculoskeletal fitness are independently associated with areal bone mineral density in children
Source: Front Physiol. 2023 Feb 13;14:1080091. doi: 10.3389/fphys.2023.1080091 (PMC9970258; doi:10.3389/fphys.2023.1080091)
Supplement: Supplementary file 1 [file DataSheet1.docx]

Supplementary Material

**Supplementary table 1.** Distribution and central tendency of the bone mineral content and areal bone mineral density variables in each body segment evaluated (160 children, phase 1).

|  | **Total** | | **Boys** | | **Girls** | |
| --- | --- | --- | --- | --- | --- | --- |
|  | **n** | **X±SD** | **n** | **X±SD** | **n** | **X±SD** |
| **BMC** |  |  |  |  |  |  |
| Total body | 159 | 1222.17±272.90 | 84 | 1234.00±253.77 | 75 | 1208.92±294.00 |
| TBLH | 159 | 882.10±242.19 | 84 | 880.00±220.05 | 75 | 884.42±266.05 |
| Upper Limbs | 159 | 130.68±34.49 | 84 | 129.44±31.00 | 75 | 132.07±38.19 |
| Trunk | 160 | 330.03±86.28 | 85 | 331.19±78.52 | 75 | 328.72±94.84 |
| Total spine | 160 | 71.95±21.55 | 85 | 70.44±18.05 | 75 | 73.64±24.92 |
| Pelvis | 160 | 126.25±34.34 | 85 | 126.6±31.36 | 75 | 125.76±37.61 |
| Lower Limbs | 160 | 424.94±130.93 | 85 | 426.11±124.49 | 75 | 423.62±138.71 |
| **aBMD** |  |  |  |  |  |  |
| Total body | 159 | 0.847±0.091 | 84 | 0.849±0.086 | 75 | 0.845±0.096 |
| TBLH | 159 | 0.717±0.098 | 84 | 0.714±0.089 | 75 | 0.722±0.108 |
| Upper Limbs | 159 | 0.559±0.075 | 84 | 0.555±0.072 | 75 | 0.565±0.078 |
| Trunk | 160 | 0.677±0.090 | 85 | 0.670±0.077 | 75 | 0.684±0.103 |
| Total spine | 160 | 0.728±0.100 | 85 | 0.718±0.081 | 75 | 0.739±0.116 |
| Pelvis | 160 | 0.715±0.106 | 85 | 0.706±0.094 | 75 | 0.724±0.118 |
| Lower Limbs | 160 | 0.831±0.124 | 85 | 0.832±0.117 | 75 | 0.830±0.133 |

n: absolute value; X±SD: average value and standard deviation; BMC: bone mineral content; aBMD: areal bone mineral density; TBLH: total body less the head.

**Supplementary table 2.** Variability Difference between sexes in physical fitness variables at each age (160 children, phase 1).

|  | **∆** | **T** | **df** | **p-value** |  | **∆** | **T** | **df** | **p-value** |
| --- | --- | --- | --- | --- | --- | --- | --- | --- | --- |
|  | **6 years-old** | | | |  | **7 years-old** | | | |
| **Sprint** | -0.63 | -2.932 | 13.5 | **0.011** |  | -027 | -1.004 | 24 | 0.326 |
| **Agility** | -1.03 | -2.424 | 24 | **0.023** |  | 0.26 | 0.602 | 25 | 0.552 |
| **Lower Limbs Power** | 20.69 | 2.636 | 25 | **0.014** |  | 9.13 | 1.266 | 25 | 0.217 |
| **Upper Limbs Power** | 22.49 | 2.161 | 25 | **0.041** |  | -18.73 | -1.017 | 25 | 0.319 |
|  | **8 years-old** | | | |  | **9 years-old** | | | |
| **Sprint** | -0.43 | -1.999 | 26 | 0.056 |  | -0.33 | -1.836 | 19 | 0.082 |
| **Agility** | -0.60 | -2.499 | 26 | **0.019** |  | -0.53 | -1.646 | 19 | 0.116 |
| **Lower Limbs Power** | 12.42 | 1.489 | 26 | 0.149 |  | 18.27 | 1.711 | 19 | 0.103 |
| **Upper Limbs Power** | 52.07 | 2.696 | 24 | **0.013** |  | 13.04 | 0.795 | 19 | 0.437 |
|  | **10 years-old** | | | |  | **11 years-old** | | | |
| **Sprint** | -0.09 | -.643 | 42 | 0.523 |  | 0.02 | 0.074 | 5 | 0.944 |
| **Agility** | -0.50 | -3.116 | 42 | **0.003** |  | 0.45 | 1.133 | 5 | 0.308 |
| **Lower Limbs Power** | 13.45 | 2.153 | 42 | **0.037** |  | 11.10 | 0.450 | 5 | 0.672 |
| **Upper Limbs Power** | 14.95 | 1.222 | 42 | 0.229 |  | 2.80 | 0.098 | 5 | 0.926 |

Bold p-values denote a significant difference (p < 0.05); ∆: mean difference; df: degrees of freedom; AGI: agility; LLP: lower limb power; ULP: upper limb power

**Supplementary table 3.** Variability difference between ages in physical fitness variables (160 children, phase 1).

|  |  | **Sprint** | | **Agility** | | **Lower Limb Power** | | **Upper Limb Power** | |
| --- | --- | --- | --- | --- | --- | --- | --- | --- | --- |
|  |  | **∆** | **p-value** | **∆** | **p-value** | **∆** | **p-value** | **∆** | **p-value** |
| **6** | 6 | - | - | - | - | - | - | - | - |
|  | 7 | -0.05 | 1.000 | -0.14 | 1.000 | -12.48 | 0.623 | 14.74 | 1.000 |
|  | 8 | 0.40 | 0.113 | 0.57 | 0.254 | -14.22 | 0.290 | -1.19 | 1.000 |
|  | 9 | 0.85 | **<0.001** | 1.01 | **0.002** | -22.79 | **0.009** | -64.51 | **<0.001** |
|  | 10 | 0.84 | **<0.001** | 1.14 | **<0.001** | -26.82 | **<0.001** | -58.55 | **<0.001** |
|  | 11 | 0.88 | **0.004** | 0.990 | 0.123 | -30.79 | **0.021** | -78.85 | **<0.001** |
| **7** | 6 | 0.05 | 1.000 | 0.14 | 1.000 | 12.48 | 0.623 | -14.74 | 1.000 |
|  | 7 | - | - | - | - | - | - | - | - |
|  | 8 | 0.46 | **0.038** | 0.71 | 0.041 | -1.74 | 1.000 | -15.93 | 1.000 |
|  | 9 | 0.90 | **<0.001** | 1.15 | **<0.001** | -10.31 | 1.000 | -79.25 | **<0.001** |
|  | 10 | 0.89 | **<0.001** | 1.28 | **<0.001** | -14.34 | 0.141 | -73.29 | **<0.001** |
|  | 11 | 0.93 | **0.002** | 1.13 | 0.037 | -18.31 | 0.821 | -93.59 | **<0.001** |
| **8** | 6 | -0.40 | 0.113 | -0.57 | 0.254 | 14.22 | 0.290 | 1.19 | 1.000 |
|  | 7 | -0.46 | **0.038** | -0.71 | 0.041 | 1.74 | 1.000 | 15.93 | 1.000 |
|  | 8 | - | - | - | - | - | - | - | - |
|  | 9 | 0.44 | 0.092 | 0.44 | 1.000 | -8.57 | 1.000 | -63.32 | **<0.001** |
|  | 10 | 0.43 | **0.022** | 0.57 | 0.107 | -12.59 | 0.312 | -57.35 | **<0.001** |
|  | 11 | 0.47 | 0.672 | 0.42 | 1.000 | -16.57 | 1.000 | -77.65 | **<0.001** |
| **9** | 6 | -0.85 | **<0.001** | -1.01 | **0.002** | 22.79 | **0.009** | 64.51 | **<0.001** |
|  | 7 | -0.90 | **<0.001** | -1.15 | **<0.001** | 10.31 | 1.000 | 79.25 | **<0.001** |
|  | 8 | -0.44 | 0.092 | -0.44 | 1.000 | 8.57 | 1.000 | 63.32 | **<0.001** |
|  | 9 | - | - | - | - | - | - | - | - |
|  | 10 | -0.01 | 1.000 | 0.12 | 1.000 | -4.02 | 1.000 | 5.96 | 1.000 |
|  | 11 | 0.02 | 1.000 | -0.02 | 1.000 | -8.00 | 1.000 | -14.33 | 1.000 |
| **10** | 6 | -0.84 | **<0.001** | -1.14 | **<0.001** | 26.82 | **<0.001** | 58.55 | **<0.001** |
|  | 7 | -0.89 | **<0.001** | -1.28 | **<0.001** | 14.34 | 0.141 | 73.29 | **<0.001** |
|  | 8 | -0.43 | **0.022** | -0.57 | 0.107 | 12.59 | 0.312 | 57.35 | **<0.001** |
|  | 9 | 0.01 | 1.000 | -0.12 | 1.000 | 4.02 | 1.000 | -5.96 | 1.000 |
|  | 10 | - | - | - | - | - | - | - | - |
|  | 11 | 0.04 | 1.000 | -0.15 | 1.000 | -3.97 | 1.000 | -20.29 | 1.000 |
| **11** | 6 | -0.88 | **0.004** | -0.99 | 0.123 | 30.79 | **0.021** | 78.85 | **<0.001** |
|  | 7 | -0.93 | **0.002** | -1.13 | **0.037** | 18.31 | 0.821 | 93.59 | **<0.001** |
|  | 8 | -0.47 | 0.672 | -0.42 | 1.000 | 16.57 | 1.000 | 77.65 | **<0.001** |
|  | 9 | -0.02 | 1.000 | 0.02 | 1.000 | 8.00 | 1.000 | 14.33 | 1.000 |
|  | 10 | -0.04 | 1.000 | 0.15 | 1.000 | 3.97 | 1.000 | 20.29 | 1.000 |
|  | 11 | - | - | - | - | - | - | - | - |

Bold p-values denote a significant difference (p < 0.05); ∆: mean difference; df: degrees of freedom; AGI: agility; LLP: lower limb power; ULP: upper limb power
